# Molecular evidence on the occurrence of co-infection with *Pichia guilliermondii* and *Wuchereria bancrofti* in two filarial endemic districts of India

**DOI:** 10.1186/2049-9957-3-13

**Published:** 2014-04-07

**Authors:** Suprabhat Mukherjee, Niladri Mukherjee, Prasanta Saini, Prajna Gayen, Priya Roy, Santi P Sinha Babu

**Affiliations:** 1 Parasitology Laboratory, Department of Zoology (Centre for Advanced Studies), Visva-Bharati University, Santiniketan- 731 235, West Bengal, India

**Keywords:** Lymphatic filariasis, *Pichia guilliermondii*, Co-infection, Polymerase chain reaction, Molecular identification

## Abstract

**Background:**

Lymphatic filariasis (LF), a vector-borne parasitic disease, is endemic in several parts of India and mostly affects the poor or those with a low-income. The disease results in huge numbers of morbidities, disabilities, and deaths every year. Association of co-infection with other pathogens makes the condition more severe. Although co-infection is becoming a growing area of research, it is yet to emerge as a frontier research topic in filarial research specifically. This study reports the occurrence of a fungal infection in a large number of patients suffering from bancroftian filariasis in two districts of West Bengal, India.

**Methods:**

Nocturnal blood samples from filarial patients containing parasites and fungus were initially co-cultured, and further the fungus was isolated and characterized. Molecular identification of the isolate was carried out by PCR-based selective amplification and sequencing of highly-conserved D1/D2 region of 26S rDNA, whereas pathogenicity was determined by amplification of the RPS0 gene. A phylogenetic tree was constructed to study the relationship between the isolate and common pathogenic yeasts. The isolate was studied for antibiotic sensitivity, whereas morphological characterization was performed by microscopic techniques.

**Results:**

The isolate was identified as *Pichia guilliermondii* and this fungus was found to exist in co-infection with *Wuchereria bancrofti* in filarial patients. The fungus showed resistance to azole antifungals, griseofulvin, and, amphotericin B, whereas significant susceptibility was evident in cases of nystatin and cycloheximide. A total of 197 out of 222 patients showed this co-infection.

**Conclusion:**

This study revealed, for the first time, that *P. guilliermondii* exists as a co-infection in microfilaraemic individuals living in a filarial endemic zone. The findings are important and have relevance to human health, especially for filarial patients.

## Multilingual abstracts

Please see Additional file 1: Multilingual abstracts in the six official working languages of the United Nations.

## Background

Lymphatic filariasis (LF), a vector-borne disease mainly caused by the filarial parasites– namely *Wuchereria bancrofti*, *Brugia malayi,* and *Brugia timori-*has become a global problem that constitutes 120 million infections per year in 81 tropical countries [1]. It is the world’s second leading cause of long-term disability. Out of the total disease burden of LF, *W. bancrofti* alone accounts for 90% and this form of LF is termed ‘bancroftian filariasis’ [2]. Currently, one-third of the affected persons are from South Asia and another third from Africa, while one-sixth of the world’s population is at risk of infection [1]. It is a disease mostly of the poor, which significantly affects this group’s ability to earn an income, and this has led to its inclusion on the list of neglected tropical diseases [1]. It results in significant economic and psychosocial impacts wherever it is endemic; disfiguring and/or incapacitating more than 40 million individuals, their families, and the endemic communities. Particularly in the Indian subcontinent, the disease affects the work time of infected patients and thereby costs the National Treasury a minimum of US$842 million per year [3]. Lymphatic Filariasis is endemic in several parts of India, including 250 districts in 20 states and six union territories (UTs), contributing 40% to the global disease burden [3]. The causative agents of LF i.e. filarial parasites live in the body cavity or tissues of vertebrate hosts where they parasitize the lymphatics, which results in the obstruction of the lymphatic vessels, incompetence, lymphostasis, lymphatic dysfunction (hydrocele and lymphedema), and interstitial fibrosis, followed by immunological dysfunction and inflammation which results in elephantiasis [4]. This disease also promotes vulnerability to opportunistic infections [5], particularly during the progression of lymphedema from chronic filarial infection. Development of elephantiasis is caused by the long-term recurrent secondary infections by opportunistic microbes [4].

Opportunistic microbial infections are very common in infectious diseases that suppress the host immune system, and promote secondary infections that are of major concern as they make the host weaker or create a life-threatening condition [6,7]. Different bacteria, viruses, and fungi, especially *Candida* yeasts, have been characterized as opportunistic pathogens [8]. The epidemiology of yeast infections is rapidly evolving as co-infection in patients suffering from primary infectious diseases [9]. Although rare, non-albicans *Candida* (NAC) *spp*. are emerging as potential opportunistic pathogens, among which *Pichia guilliermondii* (formerly known as *Candida guilliermondii* or *Meyerozyma guilliermondii*) is one of the 15 yeast species related to human diseases [9,10]. It is commonly isolated from clinical specimens such as phlegm, wounds, sputum, and blood [11]. Although *P. guilliermondii* is least pathogenic compared to the other fungi of the *Candida* family, it can still be responsible for life-threatening infections in immunocompromised hosts [9,12]. It constitutes 35–65% of all candidaemias in the general patient population and is mostly evident in cancer patients, bone marrow transplant recipients and, to a lesser degree, in intensive care unit patients, children, and surgical and HIV-positive patients [13]. As reported by Horn *et al.*[14], 1–5% prevalence of *Candida* infection is contributed by *P. guilliermondii*. The large-scale studies of candidaemia between 1999 and 2006 found that around 15% of a total of 9,717 cases were due to the *P. guilliermondii* infection [12]. Dick *et al.*[15] previously reported death due to disseminated candidiasis caused by the *P. guilliermondii* infection. Recently, this pathogen has been reported to cause infection in the knee of a patient lacking predisposing factors [16]. Occurrence of *P. guilliermondii* out of the total *Candida* isolates in different geographic locations is 1.1% in the Asia Pacific, 1.0% in Europe, 3.7% in Latin America, and 0.6% in North America [17].

Particularly for filariasis, Ormerod *et al.*[18] reported an unusual and chronic anaerobic urinary infection in the filarial patients caused by *Bacteroides melaninogenicus*, *B. fragilis*, *Peptococcus prevotii,* and *Propionibacterium granulosum,* passed from the abnormal lymphatics. Date *et al.*[19] reported severe lymphocytopenia, extensive mucosal candidiasis, and disseminated cryptococcosis in patients with long-standing filarial chyluria with immunological abnormalities. Recently, Metenou *et al.*[20] reviewed experimental findings on filaria/mycobacteria or filaria/*Plasmodium* co-infections in filarial patients. Although studied less, fungal infections are believed to cause problems in LF such as edema-causing skin folds and skin tears [21]. However, occurrence of *P. guilliermondii* has neither been reported from India nor from the peripheral blood stream of any microfilaraemic patient. This study reports, for the first time, the co-infection of *P. guilliermondii* with *W. bancrofti* in the blood of microfilaraemic patients living in the filaria endemic zone in West Bengal, India.

## Methods

### Study area and population

This study was conducted in two rural districts, namely Bankura (23° 14′ N and 87° 07′ E) and Birbhum (24° 35′ N and 88° 1′ 40″ E), in West Bengal, India. These regions were previously reported as endemic for lymphatic filariasis (LF), with more than 14% prevalence [22,23]. The study was approved by the Human Ethical Committee of the Sub-Divisional Hospital, Bolpur, West Bengal, India, and by the Institutional Ethics Committee of the Visva-Bharati University, Santiniketan- 731 235, West Bengal, India. Before taking blood samples, written consents were obtained from normal and infected persons.

### Blood samples

This study was carried out on the population residing in a filarial endemic region and suffering from bancroftian filariasis. The study originated and was carried out while LF was being studied for its prevalence and a clinical trial was being conducted by Gayen *et al.*[22,23]. As reported previously [22,23], all filarial patients were screened for the presence of microfilaria of *W. bancrofti* in microscopic preparation from finger-prick blood samples, which formed a thick film over a glass slide and was followed by Giemsa staining. Initially, the study involved the entire population mentioned in the LF prevalence study conducted between 2006 and 2008 by Gayen *et al.*[22,23]. Later, a total of 62 individuals from 31 endemic villages from the districts of Bankura and Birbhum were again studied between 2011 and 2012. All the patients had high circulating microfilariae (mf) count of 90 ± 5 per 20 μl of nocturnal blood. Nocturnal blood samples were collected at random from two apparently healthy microfilaraemic patients, irrespective of sex and age, from each village. In addition, blood samples were also collected from 10 endemic normal individuals (five male and five female; age 30 ± 2), who had no mf in their nocturnal blood samples or circulating filarial antigen, but who resided in the same geographical location. During blood sampling, aseptic conditions were maintained in the laboratory to avoid contamination.

### Isolation and co-culture of the parasite and yeast

Nocturnal blood samples (5 ml) were randomly collected from the patients with or without *P. guilliermondii,* but who screened for infection with *W. bancrofti* mf. Heparinized blood samples were diluted (1:1) with chilled phosphate buffered saline (PBS, 10 mM sodium phosphate buffer, 0.9% saline, pH 7.0) and centrifuged at 5,000 rpm for five minutes at 25°C to pellet down the mf. The pellet was suspended in 2 ml of culture medium containing RPMI-1640 supplemented with 10% fetal bovine serum (FBS), 1% glucose, 100 U/ml penicillin, 100 μg/ml streptomycin, and 0.25 μg/ml amphotericin B, and cultured at 37°C with 5% CO_2_ for 10 minutes in a CO_2_ incubator.

### Isolation and pure culture of *P. guilliermondii*

From the *P. guilliermondii-W. bancrofti* co-culture, 200 μl of spent medium was inoculated in fresh medium and further cultured for 48 hours at 37°C. Next, 10 μl of primary culture was retrieved and observed under a phase contrast microscope for presence of fungus (Dewinter, Italy). The inoculums from the medium were cultured and maintained in said medium until pure culture was achieved in the yeast specific medium. From the primary culture, 100 μl of inoculums were added to the pure culture medium comprising modified YPD agar containing 2% peptone, 1% yeast extract, 2% dextrose, and 6% FBS [24], and cultured at 32°C for 48 hours. After 11 consecutive sub-cultures, pure yeast colonies were obtained and maintained for further studies.

### DNA isolation, PCR amplification, and DNA sequencing

Existence of *P. guilliermondii* among filarial patients was initially observed during the PCR diagnosis of *W. bancrofti* using *Wolbachia* (an endosymbiont of *W. bancrofti*) specific primers, which are listed in Table 1. Following the method described by Gayen *et al.*[22], DNA was isolated from the mf rich blood samples. Molecular identification of the *W. bancrofti* infection was determined by PCR-based amplification of filaria and *Wolbachia* specific genes (see Table 1). Multiple bands obtained from the amplification of the Wolbachial gene (wsp) were sequenced and showed the existence of metagenomic DNA of *P. guilliermondii* (detail is given in the Results section)*.*

**Table 1:** **Oligonucleotide primers used for the PCR-based identification of the fungal isolate and *Wolbachia***

**Gene**	**Oligonucleotide sequence**	**Source/Reference**
Filaria-specific 28S rRNA (BD1A)	Forward: 5′ATGAAAGGCGTTGATATATAG3′	Gayen *et al*., [22]
	Reverse: 5′GCAAGCCATGCAAGCGTTGAG3′	
Wolbachia 16S rRNA-specific	Forward (FIL-5): 5′ TGAGGAAGATAATGACGG3′	Smith and Rajan, 2000 [25]
	Reverse (FIL-6): 5′CCTCTATCCTCTTTCAACC3′	
WSP int	Forward: 5′TAGCTTACTACATTCGCTTGCA3′	Bazzocchi *et al*., 2000 [26]
	Reverse: 5′CCAACTAGTGCCTATAAAGAAC3′	
26 s rDNA	Forward (NL1): 5′GCATATCAATAAGCGGAGGAAAAG3′	O’Donnell, 1993 [27]
	Reverse (NL4): 5′GGTCCGTGTTTCAAGACGG3′	
RPS0	Forward: 5′CTTGGGTTCCAAGAACGTGATT3′	Martinez *et al*., [24]
	Reverse: 5′CTTCAGCATTCCTCAGCCTTGGA3′	

In order to confirm the initial identification of the fungal isolate as *P. guilliermondii*, yeast cells were grown overnight on modified YPD broth at 32°C, with shaking at 400 rpm. DNA was extracted and purified following the procedures of Lee and Taylor [28]. Identification of the fungal isolate was carried out through the amplification of the highly-divergent D1/D2 region of 26S rRNA of the yeast using the conserved primers (NL-1 and NL-4) described previously [29]. All the primers along with their nucleotide sequences and T_m_ are summarized in Table 1. PCR was carried out for the amplification of 26S rRNA gene of the fungal isolate under optimum amplification conditions using a Gradient Thermocycler (Bio-Rad laboratories, USA). Conditions applied for the PCR amplification were as follows: initial denaturation at 94°C for four minutes followed by 30 cycles of 30 seconds at 94°C, 30 seconds at 48°C, one minute at 72°C, and a final elongation step of five minutes at 72°C. The mixture contained 5 pmoles of each primer, 5 nmoles of dNTPs, 1.5U Taq polymerase in its 1X buffer, and, 50 ng of DNA from the yeast isolate in a final volume of 50 μl. Next, 2 μl was checked in 1.8% agarose gel electrophoresis in a 1X Tris-Borate-EDTA buffer. After running for two hours at 120 V, the gel was stained in an ethidium bromide bath, de-stained in water, and observed under UV illumination using Gel Doc™ (Bio-Rad, USA). The PCR amplicon obtained from the amplification of D1/D2 region of the 26S rDNA gene using yeast specific NL-1 and NL-4 primers was purified by gel extraction and subjected to automated DNA sequencing using the commercial service available at Xcelris genomics (Xcelris Labs Ltd., Ahmedabad, India). All the nucleotide sequences were submitted to GenBank (http://www.ncbi.nlm.nih.gov) using Sequin software.

### *In silico* phylogenetic analysis

The isolated organism was formally identified by a BLASTn search using the sequenced DNA against sequences in existing DNA databases of reported organisms compiled by the NCBI (http://www.ncbi.nlm.nih.gov). The BLASTn program (http://www.ncbi.nlm.nih.gov) was used to align 26S rDNA sequence of the isolate to find the closest homologs. A total of 16 different yeast species, including *P. guilliermondii*, were subjected to pair wise and multiple sequence alignment using the ClustalW program [30]. An unrooted phylogenetic tree was constructed for 26S rDNA using the maximum parsimony method employing the subtree pruning and regrafting (SPR) algorithm [31] provided in the software package MEGA 5.1 [32]. The SPR algorithm operated with search level 0 in which the initial trees were obtained by the random addition of sequences (10 replicates). Included codon positions were 1st + 2nd + 3rd + Noncoding; the positions containing gaps and missing data were eliminated. Furthermore, reliability of the maximum parsimony tree was tested by the bootstrap method (500 replicates), provided by the software package MEGA 5.1 [32].

### Screening for pathogenicity

Pathogenicity of the fungal isolate was determined by the PCR-based detection of RPS0 gene previously described by Martinez *et al.*[24]. Selective amplification of the RPS0 gene was carried out using the primers (see Table 1) designed for the RPS0 exon region for *P. guilliermondii* by Martinez *et al.*[24].

### Scanning electron microscopy (SEM)

Yeast cells were isolated by centrifugation and processed for SEM analysis following the method described by Hayat [33], with some modifications. In brief, cell pellets were suspended in cold phosphate buffer (50 mM, pH 7.0) and incubated after adding 2.5% glutaraldehyde (Merck, Germany) for 24 hours at 4°C for fixation. Fixed cells were dehydrated by graded ethanol (10–99.9%; Merck, Germany) at room temperature (25 ± 5^ᵒ^C) and coated with 99.9% pure gold using a sputter gold coater, scanned and observed using a Scanning Electron Microscope (Hitachi, Japan).

### Determination of antibiotic sensitivity

The antimicrobial profile of the isolated fungal strain was determined by the disc diffusion method on modified YPD agar plates (with serum) using freshly-prepared inoculums from the exponential phase of the growth. Antibiotic susceptibility of the yeast isolate was tested using azole antifungals (fluconazole, clotrimazole, voriconazole, posaconazole and miconazole), griseofulvin, amphotericin B, nystatin, nikkomycin Z, terbinafine, caspofungin, and cycloheximide. The antimicrobial discs were applied on the fungal culture plates and incubated at 32°C for 24 hours. The inhibition zone appearing around each disc was measured and the sensitivity was determined from the zone diameter appearing on the plate following CLSI (formerly NCCLS) guidelines [34]. A zone with diameter of less than 13 mm in the presence of an antimicrobial was interpreted as resistant, a zone with a diameter of 15–16 mm was considered as having intermediate sensitivity, and a clear zone with a diameter of 17 mm or more indicated a high degree of sensitivity towards that antimicrobial. All the data were representative of five independent observations.

### Prevalence of *P. guilliermondii* among microfilaraemic patients

Prevalence is a common epidemiological measure of any infectious disease in a population. Since we have studied the prevalence of LF caused by the *W. bancrofti* infection in the two districts of West Bengal [22], it was interesting to study the prevalence of this typical fungal infection in the microfilaraemic patients under investigation. We have investigated the occurrence of the *P. guilliermondii* infection among 222 microfilaraemic patients (infected with *W. bancrofti*) in 32 different filarial endemic villages in two rural districts (Birbhum and Bankura) of West Bengal. The % prevalence was calculated by dividing the number of persons who harbor the *W. bancrofti-P. guilliermondii* co-infection by the number of microfilaraemic individuals who have *W. bancrofti* but not *P. guilliermondii*, and then multiplying this number by 100. To study the prevalence of *P. guilliermondii*, both molecular identification and culture analysis were employed to avoid artifact in the result.

### Statistical analysis

Statistical analysis was performed using GraphPad Prism 5.0 and Minitab 16 in the Windows environment. The difference between experimental data were analyzed by two-way ANOVA and further confirmed by the Tukey’s test.

## Results

### Molecular identification of *P. guilliermondii*

**Figure 1:**
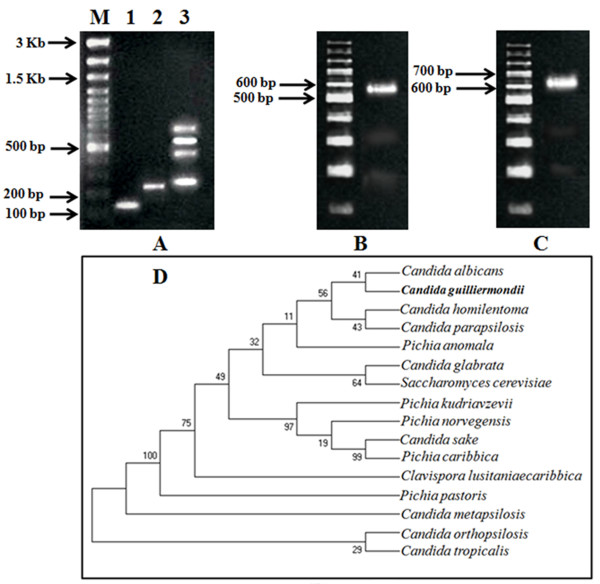
**Molecular identification and phylogeny of *P. guilliermondii *isolated from filarial patients. A**. Agarose gel showing PCR amplicon of 18S rDNA of *W. bancrofti*, Wolbachial 16S rDNA, and *Wolbachia* surface protein. M: DNA ladder (100 bp to 3 kb), L1: filarial 28S rDNA specific amplicon, L2: Amplified 16S rDNA of *Wolbachia*, L3: wsp-int specific amplicon. **B**. Agarose gel representing the PCR amplicon of D1/D2 region of 26S rDNA of *P. guilliermondii*. **C**. Agarose gel showing PCR amplicon of *P. guilliermondii* DNA amplified with RPS0 gene specific primers. **D**. Maximum Parsimony analysis of the phylogenetic relationship between *P. guilliermondii* and some common yeast species. The numerals given on the branches of the tree indicate % bootstrap value (BV). In the tree, BV values less than 50 were considered low. Figure **A**, **B**, and **C** are the representative of experiments carried out in triplicates and repeated at least five times.

Identification of infectious agents through PCR diagnosis provides a feasible option for the correct identification of such pathogens, as well as of the accurate therapeutic strategies that can be used to treat them. The result of PCR and DNA sequencing based molecular identification studies revealed the fungal isolate as *P. guilliermondii* (see Table 2 and Figure 1). As we have mentioned in the introduction, during identification of *Wolbachial* endosymbiont by conventional PCR-based amplification of the wsp gene (a routine technique for determining the *W. bancrofti* infection), almost in every case three different amplicons (size: 252, 504, and 630 bp), apart from the wsp amplicon (590 bp), were evident in the agarose electrophoresis (see Figure 1A). These amplicons were sequenced and subjected to a similarity search using the BLASTn program that showed 99% similarity with 5.8S rDNA, 99% of partial 26S rDNA, and NTS1 (Ribosomal Non Transcribed Spacer 1), 5S rRNA gene and partial NTS2 of *P. guilliermondii* (see Table 2)*.* These preliminary results have prompted us to identify the fungus from its pure culture. The PCR amplification of isolated DNA with yeast specific universal primers produced an amplicon of highly-conserved D1/D2 region of 26S rDNA of approximately 540 bp size that primarily identified the organism as yeast (see Figure 1B and Table 2). This finding was verified thrice and the amplicon was sequenced. BLASTn analyses of nucleotide sequences obtained from DNA sequencing using yeast specific universal NL-1 (forward) and NL-4 (reverse) primers showed a high degree (≥ 99%) of similarity with yeast specific 26S rDNA for *P. guilliermondii* (*C. guilliermondii* or *M. guilliermondii*), available in NCBI database (GenBank accession no: JX649967.1) (Table 2). Moreover, pair wise alignment of the sequenced DNA with reference 26S rDNA sequence of *Pichia sp.* (GenBank accession no: JX951173) showed 99% and 100% identity, respectively, for forward and reverse primer-based sequenced DNA (data not shown). The partial sequencing of the D1/D2 region of 26S rDNA further identified the organism as *Pichia guilliermondii* and was registered in GenBank with the accession no. KC771883 (see Table 2). As demonstrated in Figure 1C, an amplicon size of approximately 610 bp of RPS0 indicated the pathogenicity of the isolate. Since the primers were designed from the RPS0 exon, one can therefore conclude that the RPS0 gene exists as exon in *P. guilliermondii*. Interestingly, the intensity and pattern of pathogenicity of *P. guilliermondii* is different from *C. albicans*, which might be due to the dissimilarity in the RPS0 gene sequence [24].

**Table 2:** **DNA sequencing based identification of *P. guilliermondii***

**Amplicon size**	**Identified *P. guilliermondii *** **sequence/metagenome**	**GenBank accession no.**
630 bp	NTS1, 5S rRNA gene, and partial NTS2. 96% identity with reference sequence (accession no: FN554234.1).	KC970159
540 bp	D1/D2 region of 26S rDNA. 99.9% similarity with the reference nucleotide sequence (accession no: JX649967.1)	KC771883
504 bp	83% identical with the reference partial mRNA sequence of hypothetical protein (accession no: XM_001482915.1).	KC970158
252 bp	61% similarity with the reference partial mRNA sequence of hypothetical protein (accession no: XM_001486685.1).	KC970157

Major pathogenic *Candida spp.* including our isolate along with *S. cerevisiae*, which were positioned distinctly in the phylogenetic tree based on 26S rDNA, resembled *C. albicans* and *C. tropicalis,* respectively, as the closest and the most distant neighbor of *P. guilliermondii* (see Figure 1D). The phylogenetic tree out of the six most parsimonious trees (length = 141) is shown in Figure 1D. The consistency index is 0.773050 (0.757576), the retention index is 0.853881 (0.853881), and the composite index is 0.660093 (0.646880) for all sites and parsimony-informative sites (in parentheses) (see Figure 1D). Bootstrapping values (% BVs) suggested that the genus *Candida* is not a monophyletic group and the species can be categorized into four monophyletic groups based on BV% (see Figure 1D). The first group (BV 56%) constitutes two species pairs in which *C. albicans/C. guilliermondii* (*P. guilliermondii*) were phylogenetically close with BV 41% (see Figure 1D). Thus, the bootstrap test also supported the result of the maximum parsimony analysis. As demonstrated by Hillis and Bull [35], BV 70% is considered to indicate well-established groups. In this tree, the pair comprising *C. sake/P. caribbica* was inferred as a well-supported monophyletic group according to the bootstrap test (BV 99%), as depicted in Figure 1D. It is also worth noting that the *C. orthopsilosis/C. tropicalis* pair appeared as a cluster with a low BV (29%) (see Figure 1D).

### Microscopic characterization of *P. guilliermondii*

**Figure 2:**
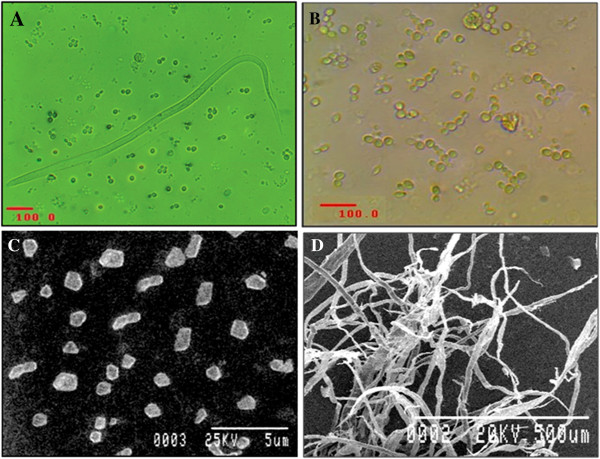
**Micrographs of *P. guilliermondii *isolated from the blood stream of microfilaraemic patient residing in the two studied filarial endemic districts in West Bengal, India. A**. Phase contrast micrograph showing *P. guilliermondii* cells with *W. bancrofti* after they have been co-cultured for a period of 24 hours. **B**. Phase contrast micrograph showing characteristic morphology of *P. guilliermondii* grown under optimum condition in pure culture. **C**. Scanning electron micrograph showing typical single cell morphology of *P. guilliermondii* grown in pure culture. **D**. Scanning electron micrograph showing fungal pseudohyphae after 72 hours of growth in pure culture on modified YPD agar.

Microscopic characterization is a conventional technique for microbial characterization. Figure 2A depicts the phase-contrast micrograph showing *P. guilliermondii* along with the mf of *W. bancrofti* in a co-culture, where both were viable. The characteristic microscopic morphology of the fungus was similar in both the co-culture and in the pure culture (see Figure 2A and B). Electron micrograph of *P. guilliermondii* showed characteristic single-cell morphology of *P. guilliermondii* (see Figure 2B) and the micrograph was similar with a previous report [36]. However, microbes from 72 hours of culture showed production of long un-branched pseudohyphae of the isolate cultivated on YPD agar (see Figure 2D) and the morphology of hyphae was also supported by a previous report [37].

### The antibiotic sensitivity profile of the isolate

**Figure 3:**
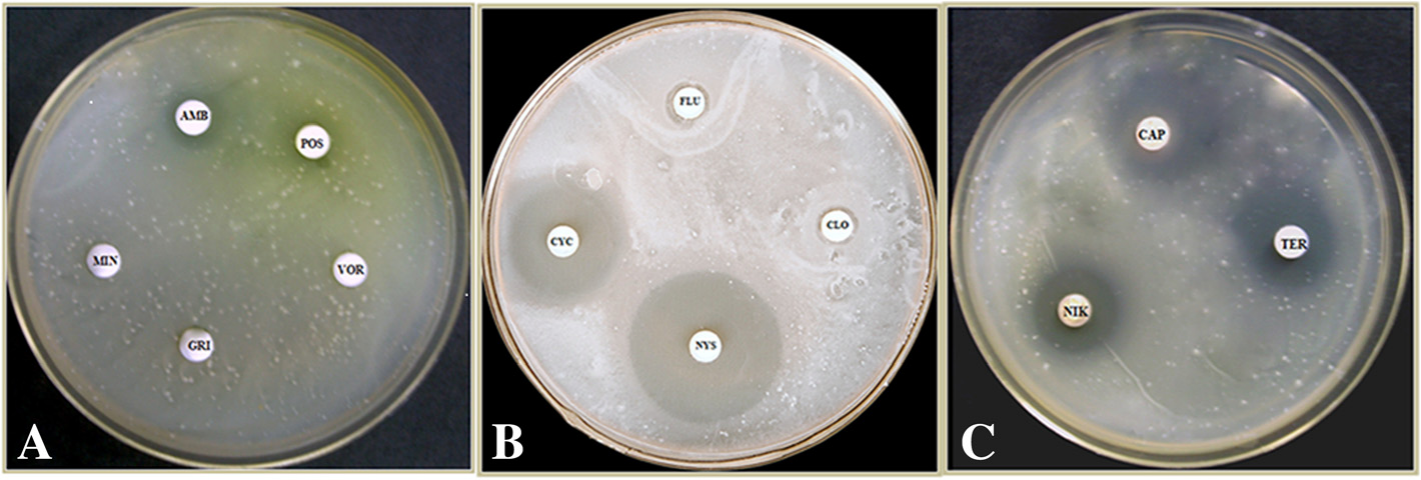
**Antibiotic sensitivity of *P. guilliermondii *grown in presence of common antifungals on YPD agar Plate. A**. *P. guilliermondii* showed resistance to voriconazole (VOR), griseofulvin (GRI), and miconazole nitrate (MIN), whereas mild susceptibility (<5 mm) was observed for amphotericin B (AMB) and posaconazole (POS). **B**. The fungal isolate showed resistance to both fluconazole (FLU) and clotrimazole (CLO), whereas detectable susceptibility was evident for nystatin (NYS) and cycloheximide (CYC), with inhibition zones of 45.0 ± 2.5 and 37.5 ± 2.3 mm, respectively. **C**. Caspofungin (CAP), nikkomycin Z (NIK), and terbinafine (TER) were found to be effective with inhibition zones of 22.5 ± 2.0, 20.0 ± 1.5, and 15.0 ± 1.5 mm, respectively. Experiments were carried out in triplicates and all the antifungal drugs were tested for susceptibility at a dose of 10 μg/ml.

The fungal isolate described in this study showed resistance to common antifungals including azoles. The antibiotic sensitivity profile of *P. guilliermondii* is given in Figure 3. It showed resistance to all azole antifungals, griseofulvin, and amphotericin B (see Figure 3A and B), whereas nystatin, nikkomycin Z, terbinafine, caspofungin, and cycloheximide were found to be effective against the isolate at a concentration of 10 μg/ml (see Figures 3B and C). These observations were corroborated by previous studies by Pfaller *et al.*[17,38]. Among those effective antifungals, nystatin and cycloheximide were the antifungals of choice with inhibition zones of 45 mm and 37.5 mm, respectively, whereas activity of other three drugs were moderate at a concentration of 10 μg/ml. MIC values for these most effective antimicrobials were 5.7 and 7.2 μg/ml, respectively for nystatin and cycloheximide.

### Prevalence of *P. guilliermondii* among microfilaraemic patients

**Figure 4:**
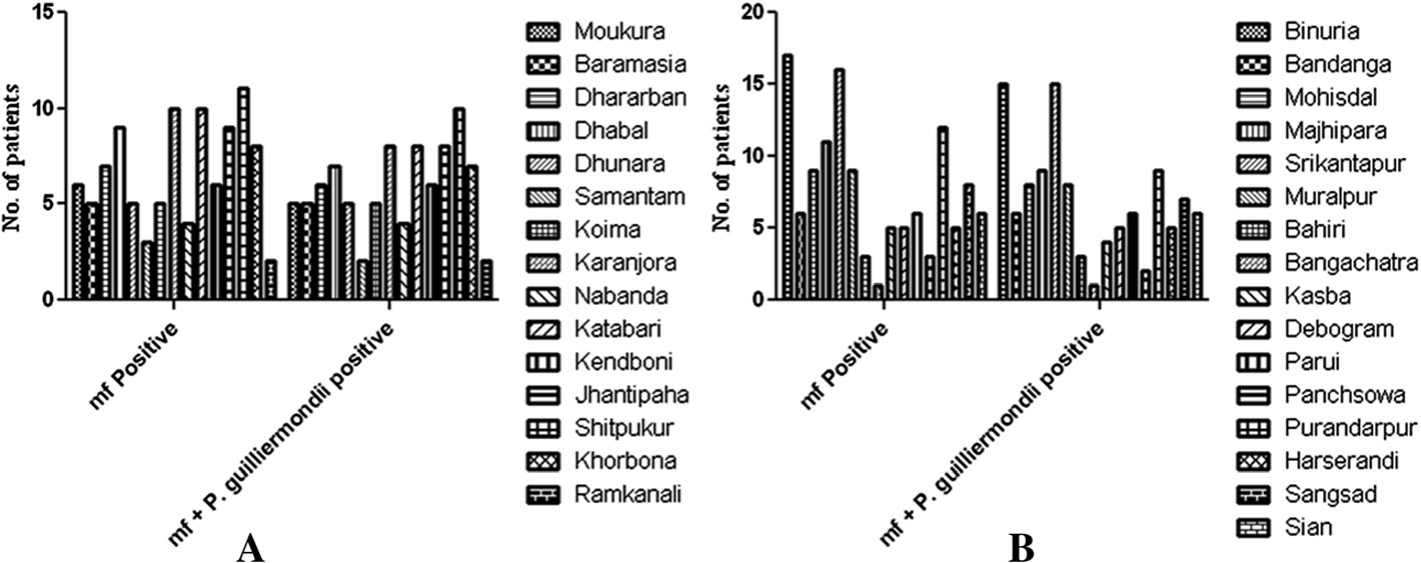
**Prevalence study of *P. guilliermondii *co-infection in microfilaraemic patients. A**. *P. guilliermondii-W. bancrofti* co-infection among the microfilaraemic patients of the Bankura district, India. **B**. *P. guilliermondii-W. bancrofti* co-infection among the microfilaraemic patients living in the filarial endemic villages of the Birbhum district, India.

We found high prevalence (88.7%) of the *P. guilliermondii* infection in microfilaraemic patients co-existing with filarial parasite. Out of the 222 microfilaraemic patients studied, 197 showed the *P. guilliermondii* co-infection, whereas the rest were devoid of the *P. guilliermondii* infection (see Figure 4). As shown in Figure 4A and B, 88 out of 100 microfilaraemic patients in Bankura and 109 out of 122 microfilaraemic patients in the Birbhum district showed parasite-fungus co-infection. The difference between the number of microfilaraemic patients and microfilaraemic patients having the *P. guilliermondii* co-infection was not statistically significant (p > 0.05). Therefore, microfilaraemic patients were likely to acquire the *P. guilliermondii* co-infection in the mentioned areas. However, individuals without a filarial infection (endemic normal) did not show any such infection. Moreover, a large number of patients did not possess any detectable symptom externally.

## Discussion

Bancroftian filariasis, caused by the *W. bancrofti* infection, covers about two-thirds of the global filarial population [39]. This form of filariasis is considered to be an important public health issue in India [39]. The disease is very ancient in the country as evident from the two oldest medical books of India namely the *Susruta Samhita* (6^th^ century BC), and the *Rug-vinischaya*, also known as the *Nidāna*, written by the physician Madhava-kara (7^th^ century AD) [3]. The endemic map constitutes 20 states and six UTs in India, where 23 million people are suffering from the disease, 31 million are mf carriers, and about 553 million are at risk of infection [3]. Previously, we have reported the prevalent status of bancroftian filariasis in the filarial endemic rural areas of the two districts, Bankura and Birbhum, in West Bengal, India [22].

Individuals suffering from bancroftian filariasis exhibit demonstrable clinical pathology that includes lymphedema, hydrocele, and elephantiasis [39]. Progressive lymphatic damage and pathology caused by the filarial parasite is due to immense tissue alteration and immunomodulation, which also promotes secondary infection by bacteria and fungus [40,41]. Previous researchers have reported bacterial and fungal co-infections in LF patients during the progression of elephantiasis [18,19,21]. However, the study of the diagnosis of fungal co-infection has been neglected to date.

Co-infection is a very important topic in relation to human health and still lacks a lot of epidemiological and experimental data. To date, there are very few researchers who have studied the co-infections associated with any form of filariasis. There has been a report of co-infection with filarial parasites and *Mycobacteria* or *Plasmodium spp.* in filarial patients, which indicated that immunomodulation and suppression of pro-inflammatory response are the principal reasons behind such co-infection [20]. However, fungal co-infection with bancroftian filariasis has not been reported to date. Our experimental and epidemiological data revealed the occurrence and molecular identification of non-albicans *Candida* (*P. guilliermondii*) co-infection in a statistically significant number of individuals suffering from bancroftian filariasis.

The identification of *P. guilliermondii*-*W. bancrofti* co-infection in microfilaraemic individuals and its prevalence were studied using PCR-based molecular diagnosis.

The method of choice for molecular diagnosis of bancroftian filariasis is the PCR-based selective amplification of the *Wolbachia* (a filarial endosymbiont) specific gene (wsp int gene). Interestingly, during the study of the LF prevalence in the mentioned districts, in most of the cases (88.7%), we observed three intense bands of 630, 504, and 252 bp, along with the wsp amplicon (590 bp). After sequencing all the bands, we came to the conclusion that, apart from wsp, the nonspecifically amplified bands belonged to a fungus i.e. *P. guilliermondii* (see Table 2). All the DNA sequences obtained from the bands shared significant similarities (up to 90%) with *P. guilliermondii*, which is synonymous to *C. guilliermondii* or *M. guilliermondii*[42]. Existence/appearance of such fungal metagenomic DNA in a number of occasions had prompted us to identify and investigate the occurrence of this organism in filarial patients. Amplification and sequencing of the D1/D2 region of the 26S rRNA gene from fungal DNA, followed by a similarity search using BLASTn, confirmed that the organism is *P. guilliermondii*. Molecular identification based on D1/D2 region of the 26S/28S rRNA gene is a reliable and robust means for identifying clinically relevant yeast isolates [43]. This PCR amplification based identification is specific, sensitive and does not involve complex and expensive equipment [24]. Sequencing of the internal transcribed regions (ITS) of the nuclear rRNA gene had especially been used to identify and discriminate between 40 species of medically important yeasts [44]. These regions have evolved slowly and show high degrees of conservation among fungi, and are thus used for molecular identification and to study the phylogenetic relationships among the isolates [43]. In this study, the phylogenetic tree based on D1/D2 region of 26S rDNA sequence of *P. guilliermondii* and its close neighbors presented a comprehensive view of their distribution, as well as their evolutionary relationship (see Figure 1D). *P. guilliermondii* was placed closely with *C. albicans* in the tree. However, the position of *P. guilliermondii*-*C. albicans* was supported by a low bootstrap value (BV% 41). In addition, selective amplification of the RPS0 gene with *P. guilliermondii* specific PCR primers further supported our finding. An amplicon of 620 bp (approximately) indicated the pathogenicity of the isolated species, and this finding corroborates with a previous report [24]. Screening of pathogenicity through a selective amplification of RPS0 has been reported as an efficient approach for the determination of pathogenicity of yeast isolates from a clinical specimen [24]. Identification and differentiation of pathogenicity of a number of *Candida spp.*, including *P. guilliermondii*, by selective amplification of RPS0 has been reported previously [45].

The antifungal susceptibility profile of pathogenic yeast varies greatly and the widespread use of antifungals might have contributed to the alteration in the species distribution through antibiotic resistance [13]. According to our study, *P. guilliermondii* tends to be resistant to common antifungals (mostly azoles). Therefore, treatment with an appropriate antifungal is required to improve survival rates in these patients. Previously, we have reported effective combinatorial chemotherapy using doxycycline (an antibacterial antibiotic) and albendazole (an anthelmintic) for the control of bancroftian filariasis in India [23]. However, neither of these drugs could eliminate this secondary infection. *In vitro* susceptibility of the isolate suggested that nystatin or cycloheximide could be the drugs to treat this fungal co-infection in filarial patients. Extensive *in vivo* studies and clinical trials are welcome to optimize the dose and duration needed to treat patients.

The high prevalence of *P. guilliermondii* in *W. bancrofti* infected individuals, inferred through first-time experimental findings, establishes the occurrence of the yeast-filarial parasite co-infection in India. Therefore, the study provides the platform to investigate the role of such co-infection in pathology and disease progression in LF.

## Conclusion

We address the PCR-based molecular identification and association of a yeast species i.e. *P. guilliermondii* co-infection in a significant number of individuals suffering from lymphatic filariasis in the districts of Birbhum and Bankura, West Bengal, India. The findings showed the presence of a new species of fungus in bancroftian filarial patients. These findings are particularly important in relation to human health, especially for filarial patients. Investigations of the *P. guilliermondii* and *W. bancrofti* co-infection in other endemic zones (involving a large number of patients), to arrive at a molecular understanding of the two species, are currently underway.

## Competing interests

The authors declare that they have no competing interests.

## Authors’ contributions

SM designed and performed all the experiments, identified *P. guilliermondii*, analyzed the data, and wrote the manuscript. NM and PR performed the antibiotic susceptibility test and culture of test samples. PS and PG carried out the prevalence study. SPS designed and supervised the study, analyzed the data, and wrote the manuscript. All authors read and approved the final manuscript.

## Supplementary Material

Additional file 1 Multilingual abstracts in the six official working languages of the United Nations. Click here for file
