# Supplementary material for: Molecular evidence on the occurrence of co-infection with Pichia guilliermondii and Wuchereria bancrofti in two filarial endemic districts of India
Source: Infect Dis Poverty. 2014 Apr 7;3:13. doi: 10.1186/2049-9957-3-13 (PMC4021973; doi:10.1186/2049-9957-3-13)
Supplement: Additional file 1 — Multilingual abstracts in the six official working languages of the United Nations. [file 2049-9957-3-13-S1.pdf]

Translation of the abstract into the six official working languages of the United Nations

دليل جزيئي على وقوع عدوى مرافقة للدودة الخيطية (الفخرية) البانكروفتية *Wuchereia bancrofti* بفطر الخميرة المعروف بـ *Pichia guilliermondii* ، في منطقتين موطنين لطفيل الخيطيات (الديدان المستديرة) في الهند.

سوبرابهات موخِرجي ، ونيلادري موخِرجي ، وبراسانتا سايني ، وبراجنا جاين ، وبريّا روي ، وسانتي ب. سينها باتو
.

الملخص.
خلفية: داء الخيطيات الليمفاوية أو داء الفيل lymphatic filariasis (LF) ، هو مرض طفيلي ينتقل عن طريق الحشرات ، وهو متوطّن في أجزاء عدة من الهند ، ويصيب غالباً الفقراء أو ذوي الدخول المنخفضة. والمرض يتسبب في أعداد كبيرة من الإصابات المرضيية ، والإعاقات ، والوفيات كل سنة.
ارتباط العدوى المرافقة بالممْرِضات الأخرى يجعل الحالة المرضية أكثر خطورة ؛ وعلى الرغم من أن مجال العدوى المرافقة أصبح مجال بحث متنامي ، إلاّ أنه بالنسبة إلى أبحاث الخيطيات بالتحديد يبدأ لتوّه كحقل بحثي جديد (يتيح مجالا لنشاط الرواد والمستكشفين). وهذه الدراسة تفيد بوقوع إصابة فطرية في عدد كبير من المرضى الذين يعانون من داء الخيطيات الليمفاوية الناتج عن الدودة الخيطية (الفخرية) البانكروفتية في منطقتين من ولاية غرب البنجال في الهند.
الوسائل: عينات ليلية من دم المريض بالخيطيات الليمفاوية محتوية على الطفيل والفطريات جرى تزريعهما معاً بداية ، ثم عُزل الفطر لاحقاً وحُددت خصائصه ؛ ثم أُجري التعرف الجزيئي على الفطر المعزول بالتضخيم والاستنساخ الانتقائيين ، وهو التعرّف القائم على تقنية الكيمياء الحيوية المعروفة بالتفاعل التسلسلي لأنزيم البوليميراز PCR ، في منطقة D1/D2 من الجين النووي 26S rDNA عالي الحفظ. ففي حين أن الإصابة المرضية تحددت بتكبير الجين RPS0 ؛ فقد أُنشئت شجرة مَحْتِد phylogenetic tree لدراسة العلاقة بين الجرثوم المعزول والخمائر الممْرِضة الشائعة. وقد دُرست حساسية الجرثوم المعزول للمضادات الحيوية ، في حين أن الخصائص البنيوية أو المعالم الشكلية أجريت بتقنيات مُجهَرية.
النتائج: تحدد الجرثوم المعزول بأنّه الفطر الخميري *Pichia guilliermondii* ، وهذا الفطر عُثر على أنه يوجد في الإصابة المرافقة للدودة الخيطية (الفخرية) البنكروفتية في المرضى المصابين بداء الخيطيات الليمفاوية. وقد أبدى الفطر مقاومة للأزول azole المضاد للفطريات ، والجريزيوفولفين griseofulvin ، والأمفوتيريسين ب amphotericin B ؛ في حين أن الحَسّاسِيّة البيّنة له قد ثبتت في حالات النيستاتين nystatin ، والسيكلوهيكسيمايد cycloheximide. وقد وُجدت هذه الإصابة المرافقة في 197 مريض من مجموع 222.
الاستنتاج/الاستدلال: كشفت هذه الدراسة لأول مرة عن أن فطر الخميرة *Pichia guilliermondii* يكون موجوداً كإصابة مرافقة في الأفرادي المصابين بالديدان الخيطية (الفخرية) البانكروفتية الذين يعيشون في نطاق موطن الخيطيات ؛ وهذه النتائج مهمة وذات علاقة بصحّة الإنسان ، خاصّة بالنسبة إلى مرضى الخيطيات الليمفاوية (داء الفيل).

Translated from English version into Arabic by Lotfi Abdolhaleem, through

TRANSLATORS
WITHOUT BORDERS

印度两个丝虫病流行区季也蒙毕赤酵母菌与班氏吴策线虫合并感染的分子证据

Suprabhat Mukherjee, Niladri Mukherjee, Prasanta Saini, Prajna Gayen, Priya Roy, and Santi P. Sinha Babu

摘要

引言：淋巴丝虫病是一种在印度多个地区流行且严重影响低收入人群健康的蚊媒传播寄生虫病。该病每年可导致大量的发病、伤残和死亡，一旦合并感染其它病原体所致后果更为严重。尽管合并感染已成为研究的上升领域，但是丝虫病研究领域尚属前沿。本研究报道了印度 West Bengal 的两个区中大量班氏丝虫病患者同时发生的真菌感染。

方法：对有合并感染的丝虫病病人进行夜间采血，先将寄生虫和真菌合并培养再分离真菌并鉴定。通过 PCR 选择性扩增和对高保守的 26S rDNA D1/D2 区测序来对分离株进行分子鉴定，而其致病性则通过 RPSO 基因的扩增来确定。构建系统发生树来研究分离株与普通致病酵母菌之间的关系。用分离株来开展抗菌性的敏感度研究，通过显微技术来进行形态学鉴定。

结果：经过鉴定，在合并感染班氏吴策线虫的丝虫病患者血中分离的产物被鉴定为季也蒙毕赤酵母菌。此真菌对唑类抗真菌药、灰黄霉素、两性霉素 B 具有抗性，然而对制霉菌素、环已酰亚胺具有明显的敏感性。222 例丝虫病患者中有 197 例合并感染者。

结论：本研究首次揭示了在丝虫病流行区微丝蚴血症者合并感染季也蒙毕赤酵母菌的存在。此发现对人体健康，尤其是丝虫病患者的健康具有重要关联。

Translated from English version into Chinese by Fu Qing, through

CHINESE CENTER FOR DISEASE CONTROL AND PREVENTION
NATIONAL INSTITUTE FOR PARASITIC DISEASES

Edited by Prof Zhou Xiao-nong (National Institute of Parasitic Diseases, Chinese Center for Disease Control and Prevention)

Preuve moléculaire de la présence d'une co-infection entre *Pichia guilliermondii* et *Wuchereria bancrofti* dans deux districts endémiques filaires en Inde.

Suprabhat Mukherjee, Niladri Mukherjee, Prasanta Saini, Prajna Gayen, Priya Roy, and Santi P. Sinha Babu

Résumé

Contexte : La filariose lymphatique (FL), une infection parasitaire à transmission vectorielle, est endémique dans plusieurs régions de l'Inde et touche principalement les pauvres ou ceux ayant de faibles revenus. L'infection résulte en un taux élevé de morbidité, d'invalidité et de décès tous les ans. Lors d'une co-infection, l'association avec d'autres agents pathogènes aggrave la condition du patient. Bien que la co-infection devienne un domaine de recherche en pleine croissance, la recherche en matière de maladie filaire plus particulièrement reste encore à émerger en terme de sujet de recherche exploratoire. Cette étude rapporte la présence d'infections fongiques chez un grand nombre de patients souffrant de filariose de Bancroft dans deux districts du Bengale Occidental en Inde.

Méthodes : Des échantillons sanguins nocturnes contenant des parasites et le champignon ont d'abord été mis en co-culture, puis le champignon a été isolé et caractérisé. L'identification moléculaire de l'isolat a été effectuée via amplification sélective par PCR et le séquençage de la région D1/D2 hautement conservée de l'ADNr 26S, alors que la pathogénicité a été déterminée par amplification du gène RPS0. Un arbre phylogénétique a été établi pour étudier la relation entre l'isolat et les levures pathogènes communes. L'isolat a été étudié pour sa sensibilité aux antibiotiques, alors que la caractérisation morphologique a été effectuée via des techniques d'imagerie microscopique.

Résultats : L'isolat a été identifié comme étant *Pichia guilliermondii,* et nous avons constaté l'existence de ce champignon lors de co-infection avec *Wuchereria bancrofti* chez des patients atteints d'une infection filarienne. Le champignon a démontré une résistance aux antifongiques azolés, à la griséofulvine et à l'amphotéricine B, mais a démontré une sensibilité importante à la nystatine et la cycloheximide. Un total de 197 patients sur 222 présentaient une co-infection.

Conclusion : Cette étude a révélé pour la première fois que *P. guilliermondii* existe en tant que co-infection chez les patients atteints de microfilarémie vivant dans des

zones filaires endémiques. Ces découvertes sont importantes et pertinentes pour la santé humaine, surtout en ce qui concerne les patients atteints de maladies filariennes.

Translated from English version into French by n_luccantoni, through

TRANSLATORS
WITHOUT BORDERS

Молекулярное свидетельство случаев коинфицирования *Pichia guilliermondii* и *Wuchereria bancrofti* в двух эндемичных филярийных районах Индии

Супрабхат Мухерджи, Ниларди Мухерджи, Прасанта Саини, Праджна Гайен, Прия Рой, Санти П. Синха Бабу

Краткое изложение

История вопроса: Лимфатический филяриатоз (ЛФ), трансмиссивное паразитарное заболевание, эндемично в нескольких частях Индии и поражает, в основном, бедные слои населения. Болезнь ежегодно приводит к огромному количеству заражений, инвалидностей и смертей. Ассоциация коинфицирования с другими патогенами усложняет течение болезни. Хотя коинфицирование становится все более активно исследуемой областью, она по-прежнему не является основной в филярийных исследованиях. Данное исследование сообщает о коинфицировании микозом среди большого количества пациентов, страдающих филяриозом Банкрофта, в двух районах Восточной Бенгалии в Индии.

Методы: Ночные образцы крови, взятые у пациентов, страдающих филяриозом, сначала содержались вместе, после чего грибки были изолированы и рассмотрены. Молекулярная идентификация изолята была проведена путем селективной амплификации на основе ПЦР и секвенирования региона высокой концентрации D1/D2 в 26S rDNA, а патогенность определялась амплификацией гена RPS0. Было создано филогенетическое древо для изучения взаимоотношений между изолятом и обычными патогенными дрожжевыми грибками. Изолят изучался на предмет чувствительности к антибиотикам, а морфологическое описание выполнялось методами микроскопии.

Результаты: Изолят был идентифицирован как *Pichia guilliermondii*, и этот грибок был найден в коинфицированных *Wuchereria bancrofti* филарийных пациентах. Грибок продемонстрировал устойчивость к азольным фунгицидам, гризеофульвину и амфотерицину Б, в то время как в случаях нистатина и циклогексимида отмечалась значительная чувствительность. Из 222 пациентов 197 имели коинфицирование.

Заключение: Данное исследование впервые установило то, что *P. guilliermondii* присутствует в случаях коинфицирования в больных микрофиларемией,

проживающих в эндемичных филярийных зонах. Результаты этого исследования очень важны для медицины, особенно для филярийных пациентов.

Translated from English version into Russian by Elena McDonnell, through

TRANSLATORS
WITHOUT BORDERS

Pruebas moleculares sobre la incidencia de coinfección de *Pichia guilliermondii* y *Wuchereria bancrofti* en dos distritos endémicos filáricos de la India

Suprabhat Mukherjee, Niladri Mukherjee, Prasanta Saini, Prajna Gayen, Priya Roy, y Santi P. Sinha Babu

Resumen

Antecedentes: La filariasis linfática (FL), una enfermedad parasitaria transmitida por vectores, es endémica en varios lugares de la India y afecta principalmente a la población pobre o de bajos ingresos. La enfermedad es responsable de una enorme cantidad de morbilidades, discapacidades y muertes cada año. El vínculo de coinfección con otros patógenos agrava la enfermedad. Aunque la coinfección está pasando a ser un ámbito de investigación creciente, aún no ha surgido como un tema ampliado en la investigación filaria en específico. Este estudio reporta la incidencia de una infección fúngica en una gran cantidad de pacientes que padecen filiariasis bancrofti en dos distritos de Bengala Occidental en la India.

Métodos: Las muestras de sangre nocturnas de pacientes con enfermedad filaria que contienen parásitos y hongos inicialmente eran cocultivadas y a continuación se aislaba y clasificaba el hongo. La identificación molecular del elemento aislado era llevada a cabo por medio de una amplificación selectiva basada en una PCR y se establecía la secuencia de una región D1/D2 altamente conservada del gen RPS0. Luego se construía un árbol filogenético para estudiar la relación entre las levaduras aisladas y las patogénas comunes. Los elementos aislados eran estudiados para determinar su sensibilidad antibiótica, mientras que la caracterización morfológica era realizada por medio de técnicas microscópicas.

Resultados: El elemento aislado fue identificado como *Pichia guilliermondii* y se descubrió que este hongo existía en coinfección con *Wuchereria bancrofti* en los pacientes con filiarisis. El hongo mostró resistencia a los antifúngicos azoles, la griseofulvina y la anfotericina B, mientras que se mostró una clara susceptibilidad considerable en casos de nistatina y cicloheximida. Un total de 197 de 222 pacientes mostraban esta coinfección.

Conclusión: Este estudio reveló por primera vez que la *P. guilliermondii* existe como coinfección en individuos con microfilaremia que habitan en una zona endémica filaria. Estos descubrimientos son importantes y tienen relevancia para la salud humana, en especial para los pacientes con filarisis.

Translated from English version into Spanish by Adriana Acevedo, through

TRANSLATORS
WITHOUT BORDERS
